# Neuroprotective Effect of Fractalkine on Radiation-induced Brain Injury Through Promoting the M2 Polarization of Microglia

**DOI:** 10.1007/s12035-020-02138-3

**Published:** 2020-10-22

**Authors:** Jiaojiao Wang, Huijiao Pan, Zhenyu Lin, Chunjin Xiong, Chunhua Wei, Huanhuan Li, Fan Tong, Xiaorong Dong

**Affiliations:** Cancer Center, Union Hospital, Tongji Medical College, Huazhong University of Science and Technology, 1277 JieFang Avenue, Wuhan, 430022 People’s Republic of China

**Keywords:** Radiation-induced brain injury, Hippocampus, Fractalkine, Microglial, CX3CR1, Inflammatory, Phagocytosis

## Abstract

Radiation-induced brain injury (RIBI) is a serious complication in cancer patients receiving brain radiotherapy, and accumulating evidence suggests that microglial activation plays an important role in its pathogenesis. Fractalkine (FKN) is a crucial mediator responsible for the biological activity of microglia. In this study, the effect of FKN on activated microglial after irradiation and RIBI was explored and the underlying mechanisms were investigated. Our study demonstrated treatment with exogenous FKN diminished radiation-induced production of pro-inflammatory factors, such as IL1-β and TNFα, promoted transformation of microglial M1 phenotype to M2 phenotype after irradiation, and partially recovered the spatial memory of irradiated mice. Furthermore, upregulation of FKN/CX3CR1 via FKN lentivirus promoted radiation-induced microglial M2 transformation in the hippocampus and diminished the spatial memory injury of irradiated mice. Furthermore, while inhibiting the expression of CX3CR1, which exclusively expressed on microglia in the brain, the regulatory effect of FKN on microglia and cognitive ability of mice disappeared after radiation. In conclusion, the FKN could attenuate RIBI through the microglia polarization toward M2 phenotype by binding to CX3CR1 on microglia. Our study unveiled an important role of FKN/CX3CR1 in RIBI, indicating that promotion of FKN/CX3CR1 axis could be a promising strategy for the treatment of RIBI.

**Electronic supplementary material:**

The online version of this article (10.1007/s12035-020-02138-3) contains supplementary material, which is available to authorized users.

## Introduction

The incidence of brain metastases has significantly increased in the past decades with improved systemic treatment [1]. A majority of patients with metastatic brain tumor have to receive radiotherapy, placing them at a high risk for RIBI, including a progressive, irreversible cognitive decline, which substantially affects the quality of life of the patients. Due to unknown mechanism of RIBI, lack of effective prevention or long-term treatment, it remains an area of active ongoing research. [2].

Although the exact pathological mechanism of RIBI remains ill-defined, studies have revealed microglia play an important role via release of inflammatory factors after radiation [3–5]. It is now well recognized that activated microglia have dual phenotypes and functional plasticity, the classical activated microglia (M1 type) or alternative activated (M2 state). The M1 phenotype can be cytotoxic, propagate tissue damage, and cause secondary injury to the brain if unregulated, while the M2 phenotype is without neurotoxic, transiently mediating neuroprotection and anti-inflammatory effects [3, 6–8]. Alternatively activated M2 microglia are divided into three subtypes—M2a, M2b, and M2c—each with different cell surface markers and distinct biological functions: M2a microglia mainly contribute to cell regeneration, whereas M2b and M2c cells participate in phagocytosis and removal of tissue debris. Microglial polarization can be regulated by transcription factors, chemokines, receptors, and interactions between microglia and other cells in the brain [9]. M1 microglia secrete pro-inflammatory cytokines such as IL-1β, IL-6, and TNF-α which were detrimental to hippocampus neurogenesis, while M2 microglia secrete anti-inflammatory cytokines such as IL-4, IL-10, and TGF-β, which is beneficial to hippocampus neurogenesis [10]. The shift between two activation states of microglia has been implicated in the responses to brain irradiation, and may also be reflective of the anti-inflammatory or pro-inflammatory responses of microglia.

FKN, an important member of the CX3C chemokine family, is secreted by neurons, vascular endothelial cells, monocytes, vascular smooth muscle cells, and T cells [11]. The FKN receptor CX3CR1 is exclusively expressed on resident microglia and considered to be the only recipient of FKN signaling in the brain. After binding to CX3CR1 on microglia, FKN mediates its effects in the brain. In pathological conditions, the dying neurons release FKN, which evoke “eat-me” signals that are recognized by microglia, resulting in phagocytosis of the dying neurons [12, 13]. Studies has revealed that FKN/CX3CR1 axis participates in the regulation of many CNS diseases including stroke, Alzheimer’s disease, multiple sclerosis, and traumatic brain injury [12]. However, the role of FKN/CX3CR1 axis remains to be fully clarified in RIBI.

Rho GTPases are molecular switches that control a wide variety of signal transduction pathways in all eukaryotic cells. They are known for their pivotal role in regulating the actin cytoskeleton, cell polarity, microtubule dynamics, and membrane transport pathways [14]. The assembly and disassembly of peripheral actin filaments can be used to promote localized changes in the structure of the plasma membrane, and phagocytosis is driven in this way to leading to phagocytosis. Not only is this important for the uptake of essential nutrients, but it is used by immature dendritic cells to sample the surrounding tissue environment [14].

In our previous studies, we demonstrated that activation of NF-κB pathway after irradiation promoted release of pro-inflammatory factors, and inhibition of microglial activation decreased expression of inflammatory cytokines and attenuated structural abnormalities of mouse RIBI model [15, 16]. However, little information has been focused on FKN/CX3CR1 axis’ effect on radiation-induced brain injury. In this study, we evaluated the effect of FKN and its interaction with inflammatory signaling on the M1/M2 phenotype changes and radiation-induced brain injury.

## Materials and Methods

### Cell Irradiation and Treatment

The mouse microglial cell line, BV-2, was maintained in the Laboratory, Center of Union Hospital, Tongji Medical College, Huazhong University of Science and Technology. Cells were cultured in Dulbecco's Modified Eagle Medium (DMEM) culture medium supplemented with 10% fetal bovine serum (FBS) at 37 °C in a 5% CO2 humidified incubator. DMEM and FBS were purchased from Gibco (Gibco, Carlsbad, USA). Cells at the logarithmic phase were used in the experiment.

Since earlier studies had reported that 10 grays (Gy) was the optimal radiation dose to activate microglia [15, 16], we used this dose for our studies. Cells were placed 100 cm away from the radioactive source. Radiation of the cells was performed with a 137Cs irradiator (Siemens, Munich, Germany) at a dose rate of 2.0 Gy/min. After 10-Gy radiation, exogenous FKN (R&D System, USA) was added at 100 ng/ml [17] to BV-2 immediately. Control cells did not receive radiation or FKN treatment. BV-2 was collected at 3-h, 6-h, 12-h, 24-h, 48-h, and 72-h post-radiation.

### Establishment of Stable BV-2 Cell Lines

Lentiviral CX3CR1 sh-RNA and the negative control constructs, which carry the puromycin resistance gene, and the corresponding virus were purchased from Huazhong Agricultural University (Wuhan, China). The titer of lentivirus was determined via serial dilution. Then, 1 × 10^8^ TU/ml lentivirus and 2 μg/ml polybrene were used to transduce BV-2 seeded in 6-well plates. Cells were incubated in 5% CO2 at 37 °C for 24 h. The medium was refreshed and cultured for another 48 h. Stable cell lines were selected by using puromycin.

### Enzyme-Linked Immunosorbent Assay

To evaluate the expression of inflammatory cytokines, the brain tissue homogenates were obtained from the hippocampus, the samples were gathered at 3 h, 24 h, 48 h, 72 h, 1 week, 2 weeks, and 4 weeks after 10-Gy CRT. The concentrations of pro-inflammatory cytokines TNF-α and anti-inflammatory cytokines IL-10 were measured by ELISA kits (Neo-Bioscience, Shenzhen, China). The absorbance at 450 nm and 630 nm was determined using a microplate reader (PerkinElmer EnSpire, Singapore). The final result was normalized to protein concentration.

### Quantitative Real-Time Polymerase Chain Reaction (qRT-PCR)

The mRNA expression level of inflammatory factors (TNF-α, IL-1β, and IL-10) were measured by real-time polymerase chain reaction (RT-PCR). Briefly, the total RNA was extracted with a Trizol reagent and subjected to complementary DNA synthesis with the TaKaRa cDNA synthesis kit (TaKaRa, Japan), according to the manufacturer’s instructions. The total PCR system contained cDNA, SYBR Green DNA polymerase, RNAse-free water, and primers. The experiment was repeated three times, and the primer sequences were designed by using Beacon Designer software package (Bio-Rad). The primers are shown as follows: TNF-α sense 5′-AGG CGG TGC CTA TGT CTCA-3′ and anti-sense 5′-GAG GCC ATT TGG GAA CTT CT-3′; IL-1β sense 5′-GAA ATG CCA CCT TTT GAC AGTG-3′, and anti-sense 5′-CTG GAT GCT CTC ATC AGG ACA-3′; IL-10 sense 5′-GCTGAAGACCCTCAGGATGC -3′ and anti-sense 5′-CCTGCTCCACTGCCTTGCT -3′; GAPDH sense 5′-TCA CCA CCATGG AGA AGGC-3′ and anti-sense 5′-GCTAAG CAG TTG GTG GTG CA-3′.

### Western Blot Analysis

Total proteins were extracted from the brain tissues and BV-2 cells by using a protein extraction kit (Pierce Biotechnology Inc., IL, USA) in accordance with the manufacturer’s protocol. Total proteins were loaded and separated on sodium dodecyl sulfate (SDS)-polyacrylamide gels and then transferred onto polyvinylidene fluoride (PVDF) membranes, rock gently for at least 1 h in blocking buffer (5% milk in TBST). Subsequently, membranes were incubated overnight with primary antibodies; namely, iNOS (1:1000, ab15323, Abcam, UK), Ym-1 (1:1000, ab192029, Abcam, UK), CD86 (1:1000, ab167720, Abcam, UK), Arg-1 (1:1000, 89007, Abcam, UK), CX3CR1 (1:500, ab8021, Abcam, UK), and GAPDH (1:2000, AC002, ABclonal, China). Next, membranes were incubated with horse-radish peroxidase labeled goat anti-rabbit or goat anti-mouse secondary antibody (1:5000, BA1054/BA1050, Boster, China) for 1 h. The ECL Western blot detection kit and an enhanced chemiluminescence system were used to examine the protein bands. The experiment was repeated three times and all measured protein levels were quantified by densitometry.

### Phagocytosis Assay

BV-2 cells were plated onto plates and were co-incubated with or without FKN after 10-Gy radiation as shown. Then the yellow-green fluorescence-labeled beads (Sigma, St Louis, MO, USA) were added for different times at 37 °C. Thereafter, the cells were washed three times with PBS to remove non-phagocytized beads and were fixed with 4% paraformaldehyde (Servicebio, Wuhan China). To stain actin filaments, cells were permeabilized with Triton X-100 (0.1%) for 5 min, incubated with fluorescent phalloidin (Servicebio, Wuhan China) for 45 min, and DAPI (Servicebio, Wuhan China) for another 5 min. Next, amounts of beads phagocytized by microglia were detected under confocal microscope (Olympus, Tokyo, Japan) and flow cytometry (Becton Dickinson, Franklin Lakes, NJ, USA). All experiments were repeated thrice.

### Animals

Four-week-old female C57BL/6 mice, including the CX3CR1 wild type mice (CX3CR1+/+, WT, CX3CR1^WT^) and the CX3CR1 homozygotic mice (CX3CR1-/-, KO, CX3CR1^KO^), obtained from the Jackson’s Laboratory (Bar Harbor, ME, USA), were maintained in specific pathogen-free animal conditions (20 °C ± 1 °C; 70% ± 10% humidity; 12 h:12 h light and dark cycle), and had free access to sterilized diet and water. All animal procedures are accordance with the National Institute of Health guidelines and were approved by the Animal Care and Use Committee at Tongji Hospital of Huazhong University of Science and Technology. Efforts were made to reduce the number of animals used and mitigate their suffering in the experiment.

### Genotyping Experiments

CX3CR1^WT^ and CX3CR1^KO^ mice were collected for PCR genotyping. Tail genomic DNA was extracted by using Mouse Direct PCR kit (Vazyme, Nanjing, Jiangsu, China). Then, the DNA lysates were subjected to PCR analysis with the following cycling conditions: 94 °C for 2 min, 10 cycles at 94 °C for 20 s, 65 °C for 15 s (− 0.5 °C per cycle decrease), and 68 °C for 10 s, then another 28 cycles at 94 °C for 15 s, 60 °C for 15 s, and 72 °C for 10 s, followed by 72 °C for 2 min. The WT allele yields a 410-bp amplicon whereas the KO allele generates a ~ 500-bp amplicon (Supplementary Fig. 1).

### Irradiation Schedule In Vivo

Mice were anesthetized by intraperitoneal injection of nembutal (1 g Nembutal dissolved in 100 ml 0.9% natrium chloride solution, 5 μl/g) and then fixed on a stereotaxic apparatus. With horizontal and vertical coordinates of the anterior fontanels, and according to the stereotactic bitmap spectra of the mouse brain, the injection location of lateral ventricle was set as follows: 2 mm after anterior fontanelle, lateral 1.6 mm of the anterior fontanelle, and 3 mm under the surface of the skull. By using an ultra-injection pump, with the speed set at 0.2 μl/min, 1 μl of FKN lentivirus solution was injected into the aforementioned site over time of 5 min.

Four-week-old female mice (13–15 g) were randomly divided into twelve groups with eight mice in each group : (1) control group (CX3CR1^WT^-Con, CX3CR1^KO^-Con), in which the mice were not subjected to CRT (cranial radiotherapy) and were injected normal saline; (2) NC (negative control) group (CX3CR1^WT^-NC, CX3CR1^KO^-NC), in which the mice were not subjected to CRT and were injected empty vector lentivirus solutions; (3) FKN (FKN lentivirus) group (CX3CR1^WT^-FKN, CX3CR1^KO^-FKN), in which the mice were not subjected to CRT but were injected FKN lentivirus solutions. (4) RT group (CX3CR1^WT^-NC (or Con) -RT mice, CX3CR1^KO^ -NC (or Con) -RT mice), in which the mice were injected empty vector lentivirus solutions (or normal saline) and received a dose of 10-Gy CRT 5 days later; (5) FKN + RT group (CX3CR1^WT^-FKN-RT mice, CX3CR1^KO^-FKN-RT mice), in which the mice received 10-Gy CRT 5 days after the FKN lentivirus solutions; After anesthesia, the mice received 10-Gy CRT as described previously with minor modifications [18].

### Morris Water Maze Test

The Morris water maze (MWM) was used to evaluate spatial learning and memory of each group at 56 days after CRT. The device consisted of a 150-cm-diameter round pool filled with water to a depth of 35 cm. The water temperature was maintained at 18–22 °C. In the target quadrant (QI) of the pool, a 15-cm-diameter escape platform was placed approximately 2 cm below the water surface. A camera was mounted directly above the center of the maze to record animal movements, and the behavioral information was analyzed using Morris water maze video tracking software (Beijing Sunny Instruments Co., Beijing, China). The mice firstly received place navigation test for five consecutive days. Mice were gently put into the water and released facing the wall from one of four quadrants in a random order. They were allowed to find the escape platform for 60 s, and the latency to escape onto the platform was recorded. If a mouse failed, it would be guided onto the platform by a stick and its latency time was recorded as 60 s. Regardless of being found on the platform or not, each mouse stayed on the platform for 10 s. Mice were trained four times a day, with inter-trial intervals of approximately 20 min. The escape latency was measured and analyzed. The day after the place navigation test, a 60-s spatial probe test was conducted with the platform removed. The times of mice crossing the platform area and the dwell time in the target quadrant where the platform was located before were recorded during the training. In addition, the swimming speed was recorded to evaluate motor function of mice for 5 days.

### Immunofluorescence Staining

The BV-2 cells were fixed in 4% paraformaldehyde for 30 min at room temperature and washed with PBS. Then the non-specific binding sites were blocked with 10% goat serum (GTX27481, Gene-Tex, USA) for 1 h at room temperature, and the samples were then incubated at 4 °C overnight with the following primary antibodies in PBS: iNOS (1:100, ab15323, Abcam, UK), Ym-1(1:100, ab192029, Abcam, UK), and F4/80 (1:50, ab6640, Abcam, UK). The following day, sections were incubated with mouse anti-rabbit IgG (1:1000, #4408/4409, Cell Signaling Technology, USA), rabbit anti-mouse IgG (1:1000, #4412/4413, Cell Signaling Technology, USA), and donkey anti-rat IgG (1:1000, A21209, Invitrogen, USA) for 1 h at room temperature. After rinsing, sections were mounted with a DAPI-containing antifade solution. Fluorescence signals were then observed under a microscope (BX63; Olympus) and a confocal microscope (Zeiss; LSM800).

### PCNA Immunohistochemistry Analysis

Mouse brains were fixed in 4% paraformaldehyde and embedded in paraffin, then sectioned at a thickness of 6 μm. For immunostaining, paraffin slides were processed through xylene, ethanol, and into water. Antigen retrieval was carried out by boiling in 10 mmol/l citrate buffer (pH 6.0). Then the sections were washed and incubated with anti-PCNA (1:150, ab18197, Abcam, UK) at 4 °C overnight. After washing, secondary biotinylated goat anti-rabbit IgG was applied at 1:200 dilutions for 30 min (Servicebio, Wuhan China). Finally, they were incubated with avidin-biotin complex kit (Servicebio, Wuhan China). Negative control sections showed no staining.

### Statistical Analysis

SPSS20.0 was used for statistical analysis. The data were expressed as the mean ± standard error of mean (SEM). All data were subjected to normal distribution (Shapiro–Wilk’s test) and homogeneity of variance tests (Levene’s test). One-way analysis of variance (one-way ANOVA), followed by post-hoc Bonferroni evaluation, was used for experiments involving multiple groups (western-blotting data, real-time quantitative PCR data, immunostaining data, flow cytometry data, and Morris water maze test results) at individual time points (for example, 6 h). Two-way analysis of ANOVA, followed by post-hoc Bonferroni evaluation, was performed for experiments across multiple time points (western-blotting data, ELISA data, Morris water maze test results). Student’s *t* test was used to evaluate differences between two groups (immunostaining data). Each experiment was repeated at least three times. *P* value < 0.05 was considered statistically significant.

## Results

### The Effect of FKN on Irradiation-Induced Microglial Activation

**Fig. 1:**
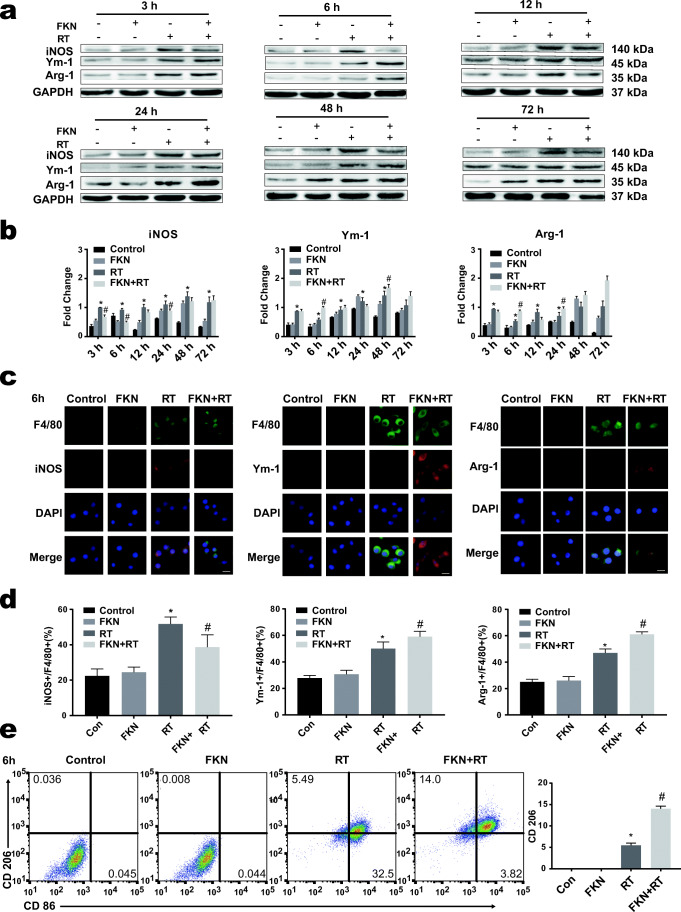
FKN promoted radiation-induced microglial M2 activation phenotypes. **a** Western blotting for the expression of M1 marker iNOS and M2 marker Ym-1 and Arg-1 in BV-2 in control, FKN (100 ng/ml), RT (10 Gy), and RT + FKN (100 ng/ml +10 Gy) groups at 3, 6, 12, 24, 48, and 72 h after 10-Gy radiation. **b** Relative quantity analysis of western blotting for iNOS/Ym-1/Arg-1. All protein expressions were normalized to GAPDH level. Values correspond to mean ± SEM (two-way ANOVA, *n* = 3 independent experiments assayed in technical triplicates (9 total samples), *P < 0.05 vs. control group; #*P <* 0.05 vs. RT-alone group). **c** Representative microphotographs of immunofluorescence staining showing expression of F4/80 (green) with iNOS or Ym-1/Arg-1(red) in BV-2 cultured with or without FKN plus 10-Gy radiation at 6 h after radiation. Scale bar = 20 μm. **d** Relative quantity analysis of immunofluorescence staining for iNOS/Ym-1/Arg-1. Values correspond to mean ± SEM (one-way ANOVA, *n* = 3 independent experiments assayed in technical triplicates (9 total samples), *P < 0.05 vs. control group; #*P <* 0.05 vs. RT-alone group**)**. **e** The BV-2 phenotype was analyzed by flow cytometry after CD86 and CD206 staining (left), and the data was processed with FlowJo (version 10.0) program (right). Values correspond to mean ± SEM (one-way ANOVA, *n* = 3 independent experiments assayed in technical triplicates (9 total samples), *P = 0.0338 < 0.05 vs. control group; *#P* = 0.001 < 0.01 vs. RT-alone group)

We firstly analyzed the effect of the FKN on the radiation-activated BV-2. The M1 and M2 microglia polarization markers were detected by western blotting. Compared to the control, the expression of the iNOS (M1 marker) and the M2 markers (Ym-1, Arg-1) were increased after 10-Gy radiation at the 3-h, 6-h, 12-h, 24-h, 48-h, and 72-h time points (Fig. 1a, b). Pretreatment with FKN (FKN + RT group) significantly increased the expression of Ym-1 and Arg-1 in BV-2, and reduced the expression of iNOS, compared with RT group, which was most pronounced at 6 h post radiation (Fig. 1a, b). Furthermore, cellular immunofluorescence assay and flow cytometry assay were performed to corroborate the results at 6 h post radiation. As Fig. 1c, d showed that, in the FKN + RT group, the fluorescence intensity of Ym-1 and Arg-1 was higher, but the fluorescence intensity of iNOS was lower, than that in RT group. The results of flow cytometry assay were consistent with the western blotting findings (Fig. 1e). Interestingly, we observed that, after radiation, the ramified BV-2 became bigger and rounder and developed the characteristic ameboid shape of activated microglia (Fig. 1c). Collectively, these data demonstrate that FKN could promote BV-2 to M2 phenotype after radiation.

### The Effect of eExogenous FKN on Radiation-Induced Inflammatory Cytokines Release

**Fig. 2:**
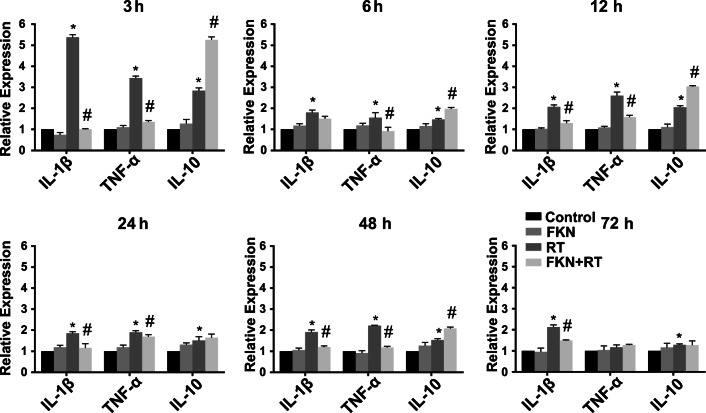
FKN inhibited radiation-induced inflammatory cytokines release Total RNA was extracted 3, 6, 12, 24, 48, and 72 h after radiation in BV-2 in control, FKN, RT, and RT + FKN groups; and mRNA expressions of IL-1β, TNF-α, and IL-10 were analyzed by real-time PCR. All gene expressions were normalized to GAPDH level. The level of control expression was fixed at 1.0. Data were expressed as relative folds compared to control. Values correspond to mean ± SEM (one-way ANOVA, *n* = 3 independent experiments assayed in technical triplicates (9 total samples), *P < 0.05 vs. control group; #*P <* 0.05 vs. RT-alone group)

After irradiation, a cascade of chemokines and cytokines are released in the tissue and augment the inflammatory response, thereby leading to possible tissue injury. The levels of pro-inflammatory cytokines (M1 markers), such as *IL-1β* and *TNFα*, were increased in BV-2 after 10-Gy radiation, while FKN treatment significantly decreased radiation-induced secretion of *IL-1β* and *TNFα* and increased the production of anti-inflammatory cytokine *IL-10* (M2 markers) at 3 h, 6 h, 12 h, 64 h, 48 h, and 72 h (Fig. 2). This result suggested FKN could alleviate radiation-induced inflammatory response.

### Exogenous FKN Promoted the Phagocytosis of BV-2 After Radiation

**Fig. 3:**
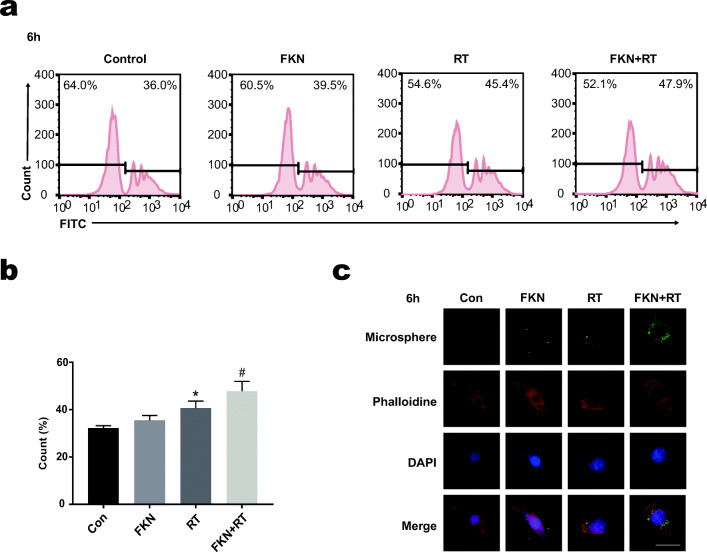
FKN promoted radiation-induced microglial phagocytic phenotype. **a** The BV-2 phagocytosis capacity was analyzed by flow cytometry after co-cultured with microsphere for 90 min in BV-2 of control, FKN, RT, and RT + FKN groups at 6 h after radiation. **b** The flow cytometry were processed with FlowJo (version 10.0) program. Values correspond to mean ± SEM (one-way ANOVA, *n* = 3 independent experiments assayed in technical triplicates (9 total samples), *P = 0.0014 *<* 0.01 vs. control group; #*P =* 0.0203 *<* 0.05 vs. RT-alone group). **c** Representative fluorescent images showing different phagocytosis of microspheres (green) with phalloidin (red) in BV-2 of control, FKN, RT, and RT + FKN groups at 6 h after radiation. Scale bar = 20 μm

Most investigators favor that microglial phagocytosis exerts a beneficial effect in repair and regeneration (M2 phenotype) [19, 20]. Fluorescent carboxylate microspheres (hereafter referred to as beads) were applied to investigate the phagocytosis capacity of BV-2. Quantification of the fluorescence of BV-2 fluorescent microspheres was done by flow cytometry analysis and immunofluorescence data. As Fig. 3a, b showed that the phagocytic capacity of BV-2 was increased after radiation, as revealed by an increase in the fluorescence of beads. And irradiated BV-2 pretreated with FKN (FKN + RT group) phagocytosed more beads than that of the RT group (Fig. 3C). The results suggested that FKN could improve the phagocytic activity of microglia.

### Effect of Interfering FKN/CX3CR1 on M2 Phenotypic Transformation of BV-2 cells

**Fig. 4:**
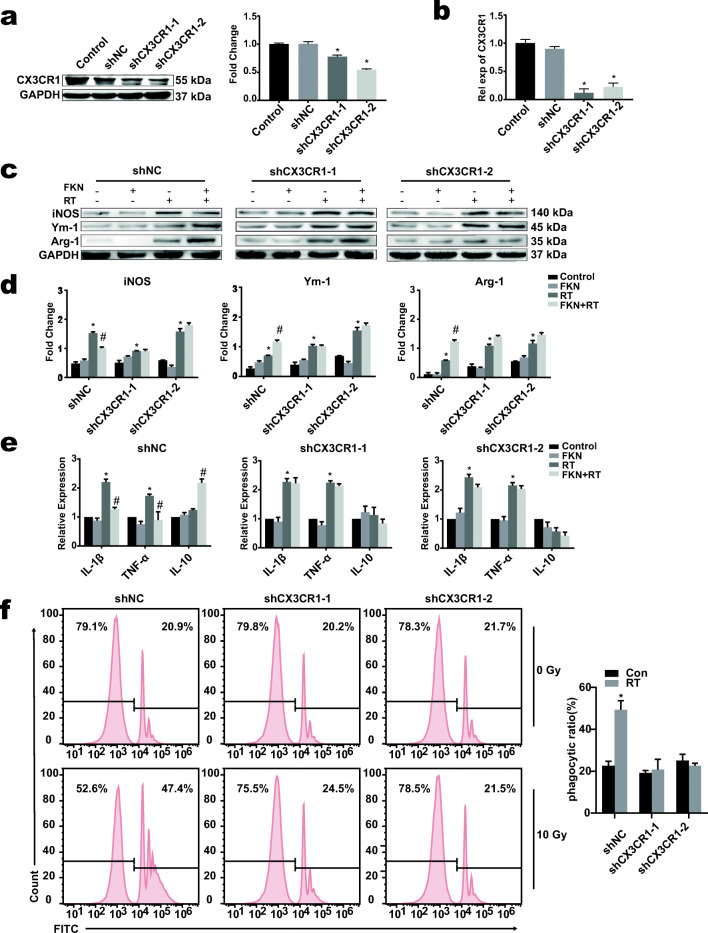
Interfering CX3CR1 partially inhibits M2 phenotypic transformation of microglia Western blotting **a** and PCR **b** showed the efficiency of lentivirus interfering the expression of CX3CR1 in BV-2. **c** BV-2 treated with lentivirus interfering the expression of CX3CR1 or NC were treated with or without FKN and 10-Gy radiation. Western blot for the expression of M1 marker iNOS and M2 marker Arg-1 and Ym-1 in BV-2 of control, FKN, RT, and RT + FKN groups at 6 h post 10-Gy radiation. **d** Relative quantity analysis of western blotting for iNOS/Ym-1/Arg-1 values correspond to mean ± SEM (one-way ANOVA, *n* = 3 independent experiments assayed in technical triplicates (9 total samples), *P < 0.05 vs. control group; #*P* < 0.05 vs. RT-alone group). **e** Total RNA were extracted at 6 h after radiation in BV-2 of each group; the mRNA expressions of IL-1β, TNF-α, and IL-10 were analyzed by real-time PCR. All gene expressions were normalized to GAPDH level. The level of control expression was fixed at 1.0. Data were expressed as relative folds compared to the control. Values correspond to mean ± SEM (one-way ANOVA, *n* = 3 independent experiments assayed in technical triplicates (9 total samples), *P < 0.05 vs. control group; #*P <* 0.05 vs. RT-alone group). **f** The phagocytosis capacity of BV-2 interfered with CX3CR1 was analyzed by flow cytometry after co-cultured with microsphere for 90 min (left). The flow cytometry data were processed with FlowJo (version 10.0) program. Values correspond to mean ± SEM (Student’s *t* test, *n* = 3 independent experiments assayed in technical triplicates (9 total samples), *P = 0.0069 < 0.01 vs. control group (0 Gy)).

Microglia have been found to respond to FKN through CX3CR1 after stimulation. To confirm the role of FKN/CX3CR1 on M2 phenotypic transformation, BV-2 cells were transduced with either lentiviral vector control or vector encoding a sh-RNA against CX3CR1. Expression of CX3CR1 in BV-2 was significantly decreased after lentiviral transduction of CX3CR1 (Fig. 4 A and B). In the sh-CX3CR1 group, the decrease of M1 type marker of iNOS and the increase of the M2 markers, Ym-1 and Arg-1, were not obviously observed after FKN and radiation treatment, compared to the sh-NC group, indicting the importance of the CX3CR1 on M2 phenotypic transformation after FKN treatment (Fig. 4c, d). Moreover, CX3CR1 knockdown abrogated the FKN-mediated increased expression levels of IL-10 and decreased TNFα and IL-1β expression levels in BV-2 cells after radiation (Fig. 4e). Furthermore, wild-type BV-2 phagocytosed more beads, after radiation, than the CX3CR1 knockdown cells. (Fig. 4f). These results suggests that exogenous FKN promoted phenotypic transformation of microglia from M1 to M2 by acting on the CX3CR1 receptors on the surface of microglia.

### Effects of FKN on Microglia Phenotype Change After 10-Gy CRT In Vivo

**Fig. 5:**
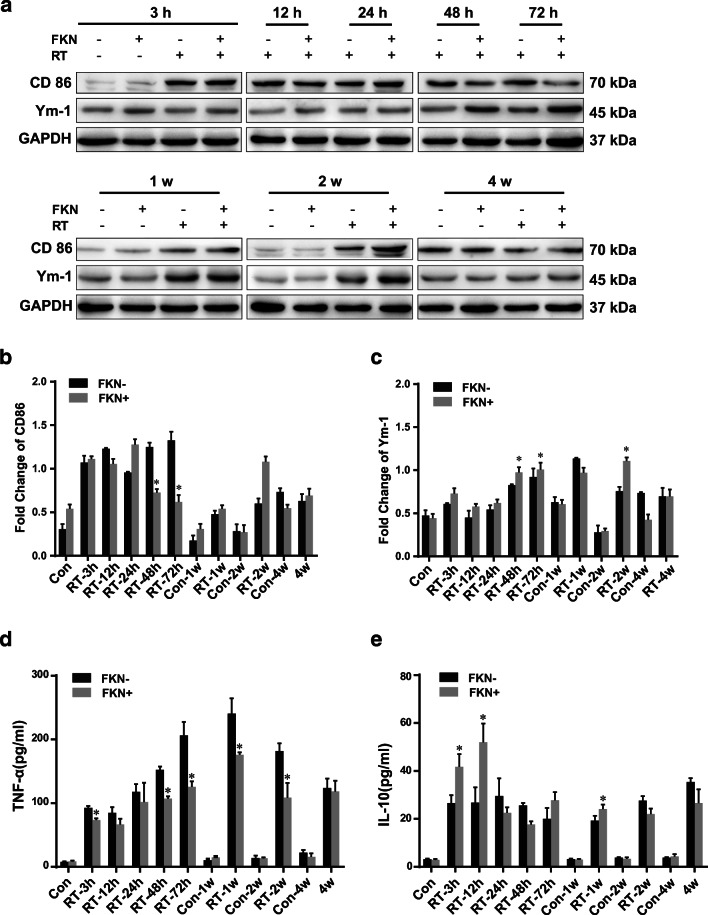
FKN promoted M2 phenotypic transformation in hippocampus of mice after CRT. **a** Protein of hippocampus from radiated mice were extracted at 3 h, 6 h, 12 h, 24 h, 48 h, 72 h, 1 week, 2 weeks, 4 weeks after CRT, protein expression of CD86, and Ym-1 were analyzed by western blotting. **b** Relative quantity analysis of western blotting for CD86. **c** Relative quantity analysis of western blotting for Ym-1. All protein expressions were normalized to GAPDH level. ELISA showed that the levels of TNF-α **(d)** and IL-10 **(e)** in radiated mice blood at 3 h, 6 h, 12 h, 24 h, 48 h, 72 h, 1 week, 2 weeks, 4 weeks after CRT. Values correspond to mean ± SEM (one-way ANOVA, *n* = 3 independent experiments with 8 independent samples (24 total samples), *P < 0.05 vs. FKN-group)

Four week-old female mice were injected with or without FKN lentivirus and subsequently received 10-Gy CRT 5 days later, as described previously. To explore whether FKN contributes to the M2 polarization of microglia in hippocampus after 10-Gy CRT, we examined the expression of M1 marker (CD86) and M2 marker (Ym-1) in the hippocampus of mice. Western blotting analysis showed that the expression of Ym-1 was increased in FKN + RT groups, compared with RT alone at 48 h or 72 h after CRT, with a simultaneous decrease in the CD86 expression (Fig. 5a–c). Furthermore, ELISA analysis showed that compared with that of RT alone, in the blood, the levels of pro-inflammatory cytokines (TNF-α) were decreased in mice of FKN + RT group, at 3-h, 48-h, 72-h, 1-week, and 2-week time points after 10-Gy CRT (Fig. 5d). Conversely, the levels of the anti-inflammatory cytokine (IL-10) increased at 3-h, 12-h, and 1-week time points (Fig. 5e). Collectively, these results indicated that FKN treatment reduced the pro-inflammatory response in hippocampus after 10-Gy CRT.

### Effects of FKN on Hippocampal Neurogenesis After RIBI

**Fig. 6:**
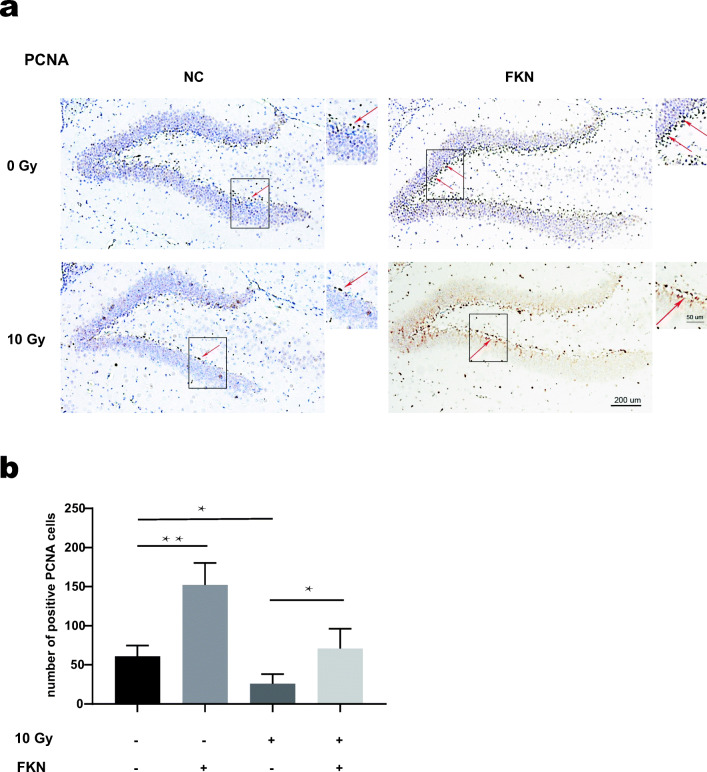
The PCNA levels of the DG in hippocampus after CRT. **a** Numbers of positive PCNA cells in the DG of hippocampus at 72 h after 10-Gy CRT, cells with dark brown (red arrows) were PCNA+ cells and cells with purple nuclei were DAPI+ cells. Scale bar = 200 μm; Scale bar = 50 μm (enlarged view). **b** The number of PCNA-positive cells in the DG was quantified. The values across the histogram represent the result of quantitative analysis of PCNA-positive cells. Values correspond to mean ± SEM (one-way ANOVA, *n* = 3 independent experiments with 7 independent samples (21 total samples), **P = 0.0007 < 0.01, control group vs. FKN group; *P = 0.0084 < 0.01, control group vs. RT-alone group; *P = 0.0036 < 0.01, RT group vs. RT + FKN group).

Above experiments demonstrated that FKN treatment promoted the M2 polarization of microglia in hippocampus after RIBI, especially at 72 h after CRT. To verify whether M2 polarization was beneficial to hippocampus neurogenesis, IF was performed to evaluate the hippocampus neural stem cells by labeling the proliferation marker PCNA (Fig. 6a). When compared with the control group, FKN lentivirus injection increased the percentage of PCNA-positive cells in the neurogenic zone of the dentate gyrus (DG) region of hippocampus. Interestingly, mice presented a reduced number of PCNA-positive cells in the DG region after 10-Gy CRT, and FKN lentivirus injection could partially reverse the CRT-induced decline in the number of neural stem cells (Fig. 6b). Collectively, these results indicated that FKN exerted a neuroprotective in RIBI mice model.

### Effects of FKN/CX3CR1 on Spatial Learning and Memory After 10-Gy CRT

**Fig. 7:**
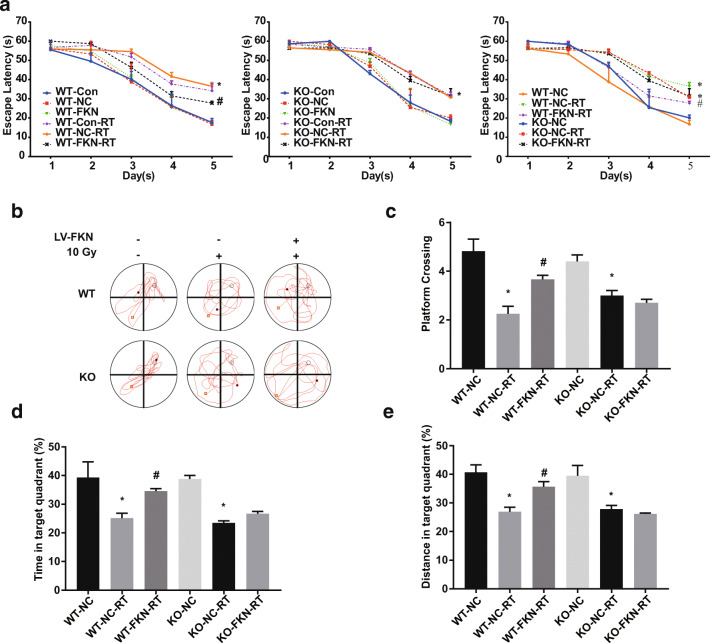
FKN slowed down the cognitive decline of mice after CRT. **a** Morris water maze (MWM) was used to confirm FKN/CX3CR1 partial restored radiated-associated cognitive impairment, escape latency for escape to a submerged platform in the training trials. **b** Representative views of the water maze for six groups during the spatial probe test. **c** The times of crossing through the location of the platform during the probe trail (left). **d** The percent of time spent in the target quadrant during the probe trail. **e** The percent of distance in the target quadrant during the probe trail (right). Values correspond to mean ± SEM (one-way ANOVA, *n* = 3 independent experiments with 8 independent samples (24 total samples), *P < 0.05 vs. control group; *#P* < 0.05 vs. RT-alone group)

To confirm the effect of FKN/CX3CR1 on radiated-associated cognitive impairment, the water maze test was performed at 6 weeks after 10-Gy CRT. CX3CR1^WT^ or CX3CR1^KO^ mice were injected with or without FKN lentivirus and subsequently received 10-Gy CRT 1 week later. In both the CX3CR1^WT^ and CX3CR1^KO^ mice, there was no significant difference in spatial learning between the Con groups, NC groups, FKN groups, RT groups, and RT + FKN groups on days 1, 2, and 3 of the water maze test (*P >* 0.05) (Fig. 7a). Conversely, the latency was observed to be longer in the RT groups compared to the NC groups or Con groups on days 4 and 5 (^*^P < 0.05) (Fig. 7a). Interestingly, the escape latency was shortened in the CX3CR1^WT^-FKN-RT group compared with the CX3CR1^WT^-NC-RT groups (or CX3CR1^WT^-Con-RT groups), especially on the day 5 (^*#*^*P <* 0.05) (Fig. 7a, left and right). On day 6, the platform was removed; the probe trial was conducted, in order to assess the spatial memory more directly. We found that CX3CR1^WT^-NC-RT mice or CX3CR1^KO^-NC-RT mice exhibited significant reduction in the number of times crossing the target (Fig. 7b, c), less time spent around the platform area (Fig. 7d), and reduced percent distance in the target quadrant (Fig. 7e), compared with the sham-irradiated group (CX3CR1^WT^-NC mice or CX3CR1^KO^-NC mice), indicating an impairment in spatial reference memory. However, when compared with CX3CR1^WT^-NC-RT mice, the number of platform crossings (Fig. 7b, c), time spent around the platform area (Fig. 7d), and the percent distance in the target quadrant (Fig. 7d) were significantly increased in CX3CR1^WT^-FKN-RT mice. Moreover, FKN lentivirus treatment was not able to enhance the behavioral performance of CX3CR1^KO^-RT mice in repeated trials; no statistical significance was observed in spatial memory function for the CX3CR1^KO^-FKN-RT mice when compared with CX3CR1^KO^-NC-RT mice (Fig. 7a–c). Altogether, these results showed that FKN lentivirus treatment remarkably ameliorated memory deficits of 10-Gy CRT mice via the FKN/CX3CR1 axis.

## Discussion

Microglial activation is considered to play an important role in the pathogenesis of RIBI, while FKN and its receptor CX3CR1 are crucial mediators in the control of microglial activity. In this study, we evaluated whether FKN could reverse activation of microglia after radiation. We found that FKN could bind to CX3CR1 on micoglial cells, attenuate RIBI through microglia phenotype modulation and the levels of inflammatory cytokines, indicating the critical role of FKN/CX3CR1 in RIBI and a potential treatment of RIBI via targeting FKN/CX3CR1 axis.

Several neuronal-mediated signals have been found to exert an anti-inflammatory action at the level of microglia. One of principal interest is the chemokine FKN, which has been considered as a novel neuro-immune regulatory protein via sending alert messages to microglia, and inhibiting its activity after stimulus. FKN is found to be expressed at the cell membrane of several neurons and can bind to and activate CX3CR1 receptors on microglia. However, the effect of irradiation on the FKN production in RIBI has never been evaluated before. In the present study, we demonstrated that FKN could promote the shift of M1 microglia towards a M2 phenotype after radiation, simultaneously improved the phagocytic function of BV-2. These results suggested that FKN might play a critical role in the development of radiation-induced inflammatory response of the brain. Therefore, investigating the regulation of its expression as well as that of its specific ligand fractalkine during RIBI is of utmost importance.

Neuroinflammation induced by radiation is characterized with significantly increase of activated microglia in the DG of the hippocampus [21]. However, the coordination of responses in the central nervous system is multifaceted and complicated. Neurobiological processes of inflammation, defense, protection, and repair involve networks of cells and molecular mediators respond to stimulus [22]. Upregulation of FKN in response to pro-inflammatory signals could further regulate the release of pro-inflammatory stimuli from microglial cell types, indicating its neuroprotective role from neurotoxicity. Cardona et al. found microglial activation induced by LPS was associated with FKN/CX3CR1 signal pathway by using the CX3CR1 receptor knockout mouse and could convert the phenotype of microglial cells from a resting state into a phagocytic and neurotoxic form [23]. In the chronic coronary insufficiency model of mice, microglia/macrophages can simultaneously express both M1 and M2 phenotypic markers on the same cell at multiple time points [24]. Such a phenomenon is also observed in other diseases such as multiple sclerosis [25]. In addition, the activated microglia population can change from the early “healthy” M2 phenotype to the “sick” M1 phenotype and exist for a long time [26–28]. In recent years, this view has changed our concept of treating brain injuries. In other words, treatment should not be limited to inhibiting activation of microglia, but should pay more attention to the ratio of microglia M1/M2 phenotype in order to reduce the risk of neuroinflammation [29, 30].

Under steady-state conditions, surveillant microglia (M0 type) monitors the nervous system, and its function depends on the contact between cells and neurons and secretion of factors to inhibit microglia activity. In neuroinflammation, microglia cells detect virus infection, toxin, injury, or stroke and activate toward M1, secreting inflammatory mediators and chemokines, participating in recruitment of monocytes, macrophages, and Th1 cells, thus amplifying the inflammatory response and promoting neuronal damage. The prospect of M2 phenotype promoting regeneration is based on the hypothesis that M2 microglia can promote the protection and repair of central nervous system nerves. The M2 spectrum may contribute to the recovery of homeostasis in the central nervous system through the secretion of growth factors, the reconstruction of extracellular matrix, the removal of debris, and the promotion of healing functions, as well as the regeneration of Treg and Th2 cells [31]. In our model, radiation-induced FKN upregulation promoted M2 phenotypic transformation of BV-2 cells after radiation and ameliorated RIBI, and blockade of FKN/CX3CR1 interaction via CX3CR1 knockdown could partially reversed FKN-mediated neuroprotective effects after radiation. These results indicated that activation of FKN/CX3CR1 axis may serve as an important anti-inflammatory signal pathway that maintains microglial cells in a quiescent state in response to irradiation. Therefore, targeting FKN/CX3CR1 receptor hyper-activation signaling pathway with specific antagonists may represent a promising strategy in the treatment of RIBI.

## Conclusions

In conclusion, the present study indicates the importance of FKN/CX3CR1 axis in the microglial activation and RIBI and suggests that targeting this signaling pathway may ameliorate radiation-induced cognitive impairment

## Electronic supplementary material

Supplementary Fig. 1 The identification of wildtype (WT) and knockout (KO) mice genetype. **(A)** Agarose gel electrophoresis were used to identify the genotype of wildtype (WT) and knockout (KO) mice. CX3CR1+/+ means wildtype (WT) mice, CX3CR1-/- means knockout (KO) mice. (PDF 266 kb)
