# Supplementary figures and images for: Neuroprotective Effect of Fractalkine on Radiation-induced Brain Injury Through Promoting the M2 Polarization of Microglia
Source: Mol Neurobiol. 2020 Oct 22;58(3):1074–87. doi: 10.1007/s12035-020-02138-3 (PMC7878270; doi:10.1007/s12035-020-02138-3)

**A**

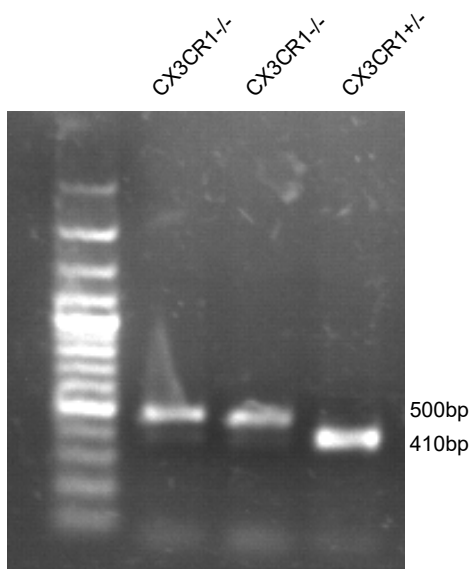

Supplement: Supplementary file 1 — The identification of wildtype (WT) and knockout (KO) mice genetype. (A) Agarose gel electrophoresis were used to identify the genotype of wildtype (WT) and knockout (KO) mice. CX3CR1+/+ means wildtype (WT) mice, CX3CR1-/- means knockout (KO) mice. (PDF 266 kb) [file 12035_2020_2138_MOESM1_ESM.pdf]
